# Prevalence and Sequence Analysis of Equine Rhinitis Viruses among Horses in Poland

**DOI:** 10.3390/v16081204

**Published:** 2024-07-26

**Authors:** Karol Stasiak, Magdalena Dunowska, Jerzy Rola

**Affiliations:** 1 Department of Virology, National Veterinary Research Institute, 24-100 Pulawy, Poland; karol.stasiak@piwet.pulawy.pl; 2 School of Veterinary Science, Massey University, Palmerston North 4442, New Zealand; m.dunowska@massey.ac.nz

**Keywords:** equine rhinitis viruses, ERAV, ERBV, horses, sequence analysis, haplotype network

## Abstract

Equine rhinitis A (ERAV) and B (ERBV) viruses are respiratory pathogens with worldwide distribution. The current study aimed to determine the frequency of infection of ERAV and ERBV among horses and foals at Polish national studs, and to determine genetic variability within the viruses obtained. Virus-specific quantitative RT-PCR assays targeting a 5′ untranslated region were used to screen nasal swabs collected from 621 horses at 16 national horse studs from throughout Poland, including 553 healthy horses and 68 horses with respiratory disease. A partial DNA polymerase gene was amplified and sequenced from the qRT-PCR-positive samples. The obtained sequences were analysed using phylogeny and genetic network analysis. None of the nasal swabs were positive for ERAV, whereas ERBV was found in 11/621 (1.78%) samples collected from 10 healthy horses and one foal affected by respiratory disease. Partial DNA polymerase gene sequence variability was correlated with individual horses and studs from which samples were collected when only Polish sequences were analysed, but there was no correlation between country of origin and ERBV sequence when Polish and international sequences were included in the network. The report presents the first detection of ERBV in Poland.

## 1. Introduction

Among all the known members of the *Picornaviridae* family, only two viruses, namely equine rhinitis A virus (ERAV) and equine rhinitis B virus (ERBV) are known to infect horses. ERAV, formerly named equine rhinovirus 1, together with bovine rhinitis viruses and foot-and-mouth disease viruses belong to the *Aphtovirus* genus [1]. ERBV belongs to the *Erbovirus* genus and has been divided into three serotypes designated ERBV1, ERBV2 and ERBV3. While ERBV1 and ERBV2 isolates are acid-labile, ERBV3 was identified to be acid-stable [2].

Infections caused by both ERAV and ERBV are widespread in equine populations worldwide and are sometimes implicated as a cause of upper respiratory disease [3]. However, in many cases, exposure to the viruses leads to seroconversion in affected horses without overt disease. The clinical signs, if present, include fever that usually lasts one to three days, anorexia, nasal discharge, cough, pharyngitis and/or dyspnea. In addition, swelling of the limbs, as well as swelling and pain of the lymph nodes of the head and neck, might be present [3]. As respiratory viruses, both infectious ERAV and ERBV are primarily found in nasal, nasopharyngeal and oral secretions [4,5,6,7]. In addition, ERAV can be detected in plasma and urine samples [8,9] and ERBV in faecal samples alone [10], indicating their potential role in urinary and gastrointestinal diseases, respectively.

Serological investigations showed that equine rhinitis viruses have a worldwide distribution including Poland [11,12]. In one of those studies, the seroprevalence of ERBV was assessed in the horse population of south-eastern Poland. However, the report was published in a national journal [12], and hence is not easily accessible to non-Polish speaking readers. Despite a recent virological survey on ERAV among Polish horses using a virus isolation test [11], little is known about the frequency of active ERAV and ERBV infections among different groups of horses throughout Poland. To the best of our knowledge, there have been no molecular investigations of equine rhinitis virus A and B in nasal secretions from different equine populations of horses in Poland.

Hence, the objective of the current study was to estimate the prevalence of infections with ERAV and ERBV among horses at selected Polish national studs and to determine genetic variability within equine rhinitis viruses.

## 2. Materials and Methods

### 2.1. Source of Samples

**Figure 1:**
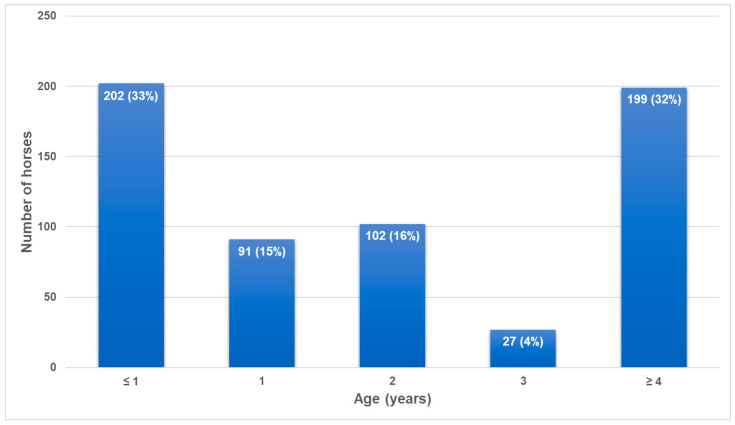
The age structure of horses sampled in the study.

A total of 621 horses from 16 national horse studs were included in the study (Table 1). Most (540/621, 86.9%) samples were collected as part of the previously published study on equine herpesviruses [13]. Breeds included Arabians (*n* = 57), Coldblood horses (*n* = 49), Hucul horses (*n* = 42), Malopolska horses (*n* = 42), Polish Konik (*n* = 198), Silesian horses (*n* = 44), Thoroughbreds (*n* = 70) and Wielkopolska horses (*n* = 119). Nasal swabs were collected into a commercial universal transport medium (Virocult, Medical Wire and Equipment Ltd., Corsham, Wiltshire, England) from each horse during a single visit to the stud by one of the authors (KS) except for studs I–III and XI. At each stud, samples were collected from all mares and foals available on the visit day. In addition, swabs were collected from approximately 10 yearlings and two-year-olds at each stud. The age structure of the sampled population is shown in Figure 1. The health status of horses was recorded during sampling. Horses were considered to be affected by respiratory disease if they demonstrated coughing or nasal discharge. Rectal temperature was not measured. Samples from horses at studs I, II, III and XI were delivered by stud veterinarians as part of a diagnostic investigation of outbreaks of respiratory disease at those studs. All swabs were submitted to the Department of Virology of the National Veterinary Research Institute in Pulawy (Poland) on ice packs within 24–48 h of collection.

### 2.2. Processing of Samples

Upon arrival, each swab was vortexed to release viruses into the transport medium and discarded. An aliquot (20–30 µL) of each medium was used to create pooled samples from five to seven horses from the same stud and age group. All samples were stored at −80 °C for further processing. Viral RNA was extracted from both pooled and individual samples using the QIAamp Viral RNA Mini kit (Qiagen, Hilden, Germany) following the manufacturer’s protocol, and eluted in a 60 µL final volume. All extraction runs included positive (ERAV isolate 393/76 or ERBV isolate 1331/3/96) and negative (transport medium) extraction controls.

### 2.3. Quantitative RT-PCR Assays

The conserved part of the 5′ untranslated region was used for the quantitative RT-PCR (qRT-PCR) assays for ERAV and ERBV, as described by Lu et al. [14]. Each virus-specific qRT-PCR reaction (25 µL) consisted of forward and reverse primers (Table 2) at a final concentration of 400 nM each, 200 nM of the probe and 5 µL of template RNA in 1× AgPath-ID™ One-Step RT-PCR buffer (Thermo Fisher Scientific, Austin, TX, USA). The samples were subjected to reverse transcription at 48 °C for 30 min, initial denaturation at 95 °C for 10 min, followed by 40 cycles at 95 °C for 15 s and 50 °C for 60 s. Negative (water) and positive (ERAV isolate 393/76 or ERBV isolate 1331/3/96) controls were included in each run. All reactions were performed using a LightCycler^®^ 96 System (Roche, Mannheim, Germany). For the ERAV and ERBV qRT-PCR, Cq values below 35.0 and 34.0 were considered positive, respectively. RNA isolated from swabs from horses with respiratory disease was tested individually, whereas RNA from the pooled samples was initially used as a template in the qRT-PCR assay. The assays were repeated with individual swabs from positive pools. All swabs from negative pools were considered negative for an ERAV/ERBV virus.

### 2.4. RT-PCR and Cloning

Viral RNA from virus-positive swabs was used as a template in a nested RT-PCR assay that amplified a product within the 3D polymerase and 3′ untranslated region, as described by Black et al. [15]. Each reverse transcription reaction as well as the first round of PCR amplification was performed using the SuperScript III One-Step RT-PCR System with Platinum Taq DNA Polymerase (Invitrogen, Carlsbad, CA, USA) in a total volume of 50 µL. The mixture consisted of 1 × reaction mix buffer (Invitrogen, Carlsbad, CA, USA), 400 nM of each outer primer (Table 2), 2 µL of SuperScript™ III RT/Platinum™ Taq Mix (Invitrogen, Carlsbad, CA, USA), and 5 µL of template RNA. Reverse transcription and the first round of amplification were performed in a Biometra Thermocycler (Biometra, Göttingen, Germany) using the following cycling conditions: cDNA synthesis at 55 °C for 30 min, pre-denaturation at 94 °C for 2 min, followed by 40 cycles of denaturation at 94 °C for 15 s, primer annealing at 55 °C for 30 s, elongation at 68 °C for 2 min and a final elongation step at 68 °C for 5 min. The second round of PCR reactions was performed in the same thermocycler using the Invitrogen Platinum Taq DNA Polymerase kit (Thermo Fisher Scientific, Carlsbad, CA, USA) and the reaction mixture (50 µL) contained 2 µL PCR product, 200 nM of inner primers, 0.2 mM deoxynucleotide mix (Sigma-Aldrich, St. Louis, MO, USA) and 1.5 mM MgCl_2_ in 1× buffer. The mixtures were initially denatured at 94 °C for 2 min, followed by 35 cycles of 94 °C for 30 s, 55 °C for 1 min and 72 °C for 1 min. All common precautions were employed to avoid cross-contamination and no false-positive was observed in negative controls. Amplified products were subjected to electrophoresis through a 1.5% GelRed stained agarose gel, purified with a GeneJET PCR Purification Kit (Thermo Scientific, Carlsbad, CA, USA) and ligated into a TOPO TA Cloning Kit For Sequencing (Thermo Fisher Scientific, Carlsbad, CA, USA) according to the manufacturer’s instructions. Ligated products were chemically transformed into Escherichia coli JM109 Competent Cells (Promega Corporation, Madison, WI, USA). Plasmid DNA was isolated from up to five separate insert-positive clones using PureLink™ Quick Plasmid Miniprep Kit (Thermo Fisher Scientific, Vilnus, Lithuania) according to the manufacturer’s instructions and sequenced with the same primers used for amplification using BigDye^®^ Terminator version 3.1 (Applied Biosystems, Vilnus, Lithuania) on a 3730xl DNA Analyzer at the Genomed (Warsaw, Poland).

### 2.5. Sequence Analyses

The forward and reverse sequences obtained from each recombinant *E. coli* colony were trimmed and assembled into consensus using BioEdit software v.7.2.5 software. Only sequences that generated good quality data were included for further analysis—those poorly aligned were removed. Next, the obtained sequences were aligned with the analogous sequences of equine rhinitis viruses deposited in the GenBank Database using the MUSCLE algorithm from the Molecular Evolutionary Genetics Analysis software package, version 11 (MEGA11). A phylogenetic tree was constructed using the maximum likelihood method with 1000 bootstrap replicates using the K2 + G + I substitution model in MEGA11. The sequence identity (%) among sequences analysed in the study was calculated using the identity matrix in BioEdit. All nucleotide sequences were used as an input file to generate a haplotype list in the DnaSP software (version 6.12.03). The sequences representative of the haplotypes for the international network were exported in nexus format with trait blocks added to represent geographical regions (country). For the Polish-only networks, additional trait blocks were added to represent the stud number and each individual horse. The median-joining haplotype networks were produced using default parameters in PopART version 1.7 (available from http://popart.otago.ac.nz, accessed on 15 May 2024). An analysis of molecular variance was carried out in PopART using the “Simple AMOVA” command to test for correlation between the population genetic structure of obtained sequences and selected traits. The strength of correlation was shown by a PhiST value, with 0 indicating no correlation and 1 indicating perfect correlation. The corresponding *p* values were generated by reference to 1000 random permutations of the input data.

### 2.6. GenBank Accession Numbers

The nucleotide sequences of Polish ERBV described in this study were submitted to GenBank under the following accession numbers: PP839207–PP839257.

## 3. Results

### 3.1. Infection with Equine Rhinitis Viruses

**Figure 2:**
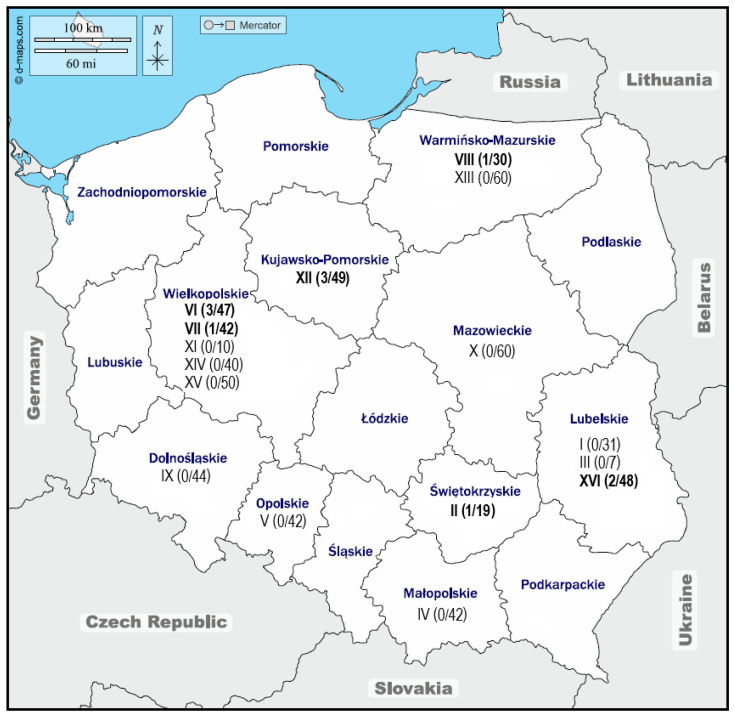
Map of Poland showing the location of the studs (denoted by Arabic numbers). The number of ERBV-positive samples/number of samples collected from each stud are shown in the brackets. Available from: https://d-maps.com/carte.php?num_car=4308&lang=en, accessed on 15 May 2024.

None of the nasal swabs tested in the study were positive for ERAV, whereas ERBV was found in 11/621 (1.78%) samples collected from horses from six different studs in five geographical regions (Figure 2). Out of 68 horses sampled with clinical signs of respiratory disease, ERBV RNA was detected in only one (1.47%) swab. The remaining 10 ERBV-positive swabs came from healthy horses (*n* = 553), which constituted 1.8% of all of those tested. The median age of horses that tested positive for ERBV was 1 year (range 0.5–3).

### 3.2. Molecular and Phylogenetic Analysis

The second-round RT-PCR products of the expected length (623 bp) were amplified from all ERBV qRT-PCR positive swabs tested. The sequence alignments of the partial 3D polymerase and 3′ untranslated region from all clones from 11 Polish ERBV viruses showed 94.3% to 100% identity at the nucleotide level and 95.6% to 100% identity at the amino acid level. The degree of identity between Polish ERBV viruses and foreign sequences was slightly lower and ranged from 92.7% to 97.4% at the nucleotide level and from 94.6 to 100% at the amino acid level. The highest genetic variability was observed between sequences from stud XVI (95.0% to 99.6% at the nucleotide level), represented by nine clones isolated from two Polish Konik horses (Table 3).

**Figure 3:**
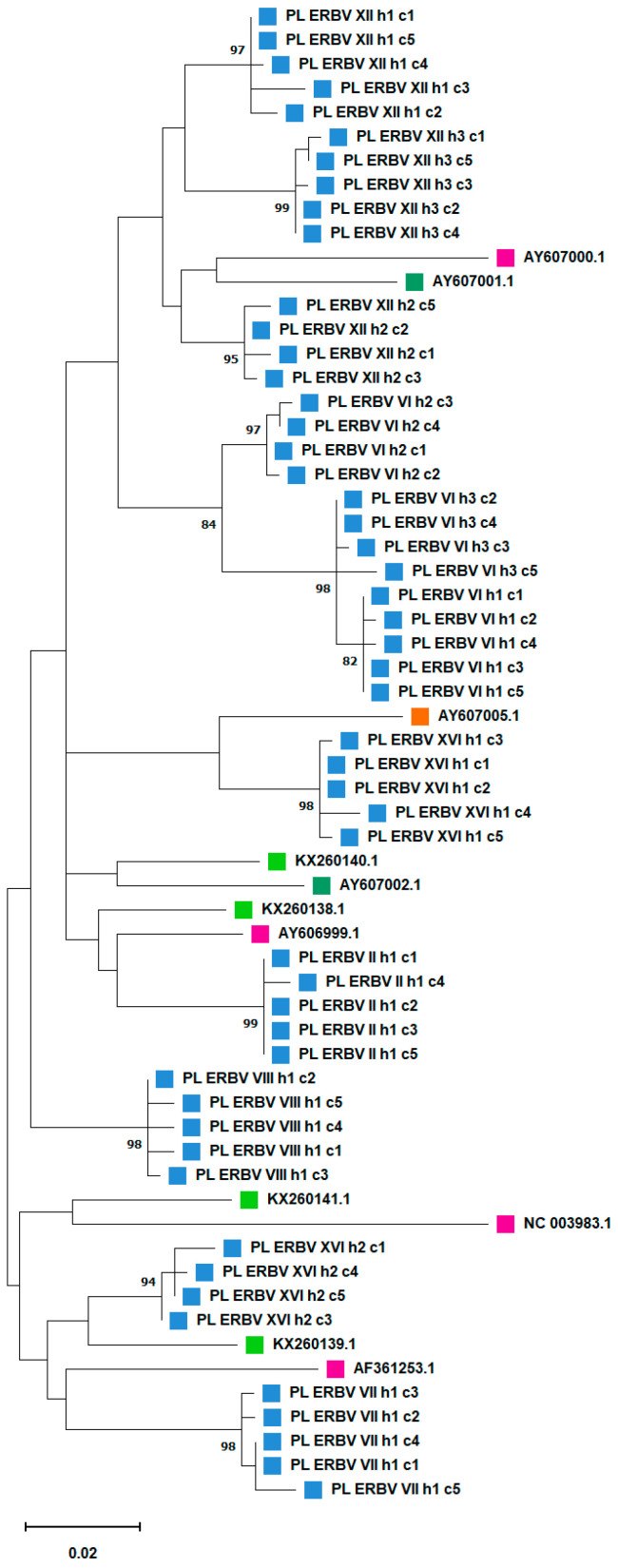
Phylogenetic tree of equine rhinitis B viruses (ERBV) based on 555 bp fragment from 3D polymerase (nt 8152-8707 in ERBV accession number NC_003983.1). The sequences used included Polish ERBV sequences of clones (*n* = 51), and international sequences (*n* = 11) sourced from GenBank. The Polish sequences obtained in the current study are labelled PL_ERBV_stud number_horse number_clone number. Accession numbers for sequences from GenBank are listed for each sequence. The evolutionary history was inferred by using the maximum likelihood method with a bootstrap value of 1000 using the Kimura 2-parameter model. The tree with the highest log likelihood (−2419.25) is shown. A discrete Gamma distribution was used to model evolutionary rate differences among sites (5 categories (+G, parameter = 0.2017)). All positions containing gaps and missing data were eliminated (complete deletion option). Evolutionary analyses were conducted in MEGA11 [16]. Coloured rectangles signify geographical origin: Poland (blue), United Arab Emirates (light green), Switzerland (pink), USA (dark green) and Australia (orange). Values greater than 80% are shown.

In general, sequences from individual horses clustered together with strong bootstrap support (Figure 3). Sequences from three horses from stud XII clustered together with ERBV sequences from Switzerland and the USA, but the bootstrap support for this grouping was low (14%). Sequences from horse 1 from stud XVI appeared to be more closely related to ERBV from Australia (AY607005) than sequences from horse 2 from the same stud, although with relatively weak (57%) bootstrap support for this grouping. Viral sequences from horse 2 from the same stud clustered with an ERBV sequence from the United Arab Emirates (48% bootstrap support).

### 3.3. Network Analysis

**Figure 4:**
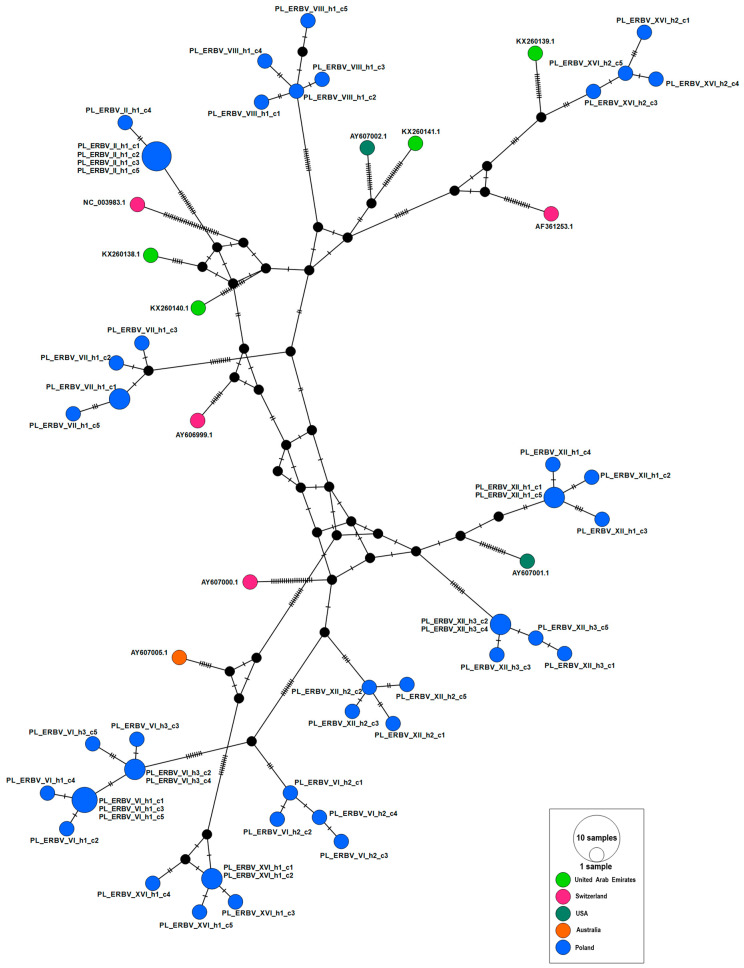
International haplotype network of ERBV based on partial (555 bp) 3D polymerase region sequences (*n* = 62) including sequences (*n* = 51) from Polish horse studs obtained in the current study and sequences from GenBank (*n* = 11). The number of nucleotide substitutions between haplotypes is represented by ticks on branches. Nodes are scaled based on the number of representative sequences and coloured based on the geographic location of origin. Small closed black circles indicate inferred nodes that are not represented among sequences included in the network.

**Figure 5:**
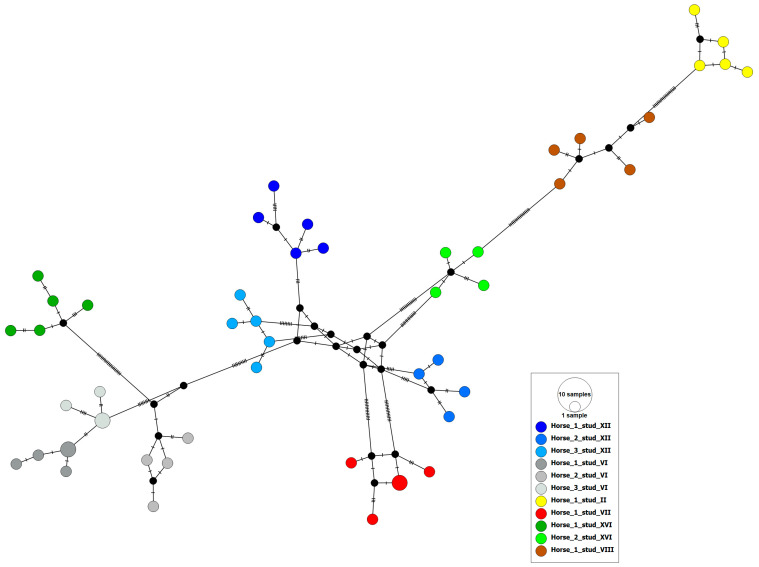
Median-joining haplotype network based on partial 3D polymerase and 3′ untranslated region of ERBV sequences (concatenated and trimmed to 621 nt) from samples originated in Poland (*n* = 51). Nodes are scaled based on the number of representative sequences and coloured based on the individual horses from which they were obtained. Horses from the same stud are shown in different shades of the same colour. Small closed black circles indicate inferred nodes that are not represented among sequences included in the network.

The 62 Polish and international ERBV partial 3D polymerase sequences (555 bp) formed 52 haplotypes. None of the haplotypes contained sequences from both Poland and international sources. Polish haplotypes (blue circles in Figure 4) were distributed throughout the international network. The clustering of ERBV haplotypes did not correlate with the country of origin (PhiST = 0.03, *p* = 0.021). The median-joining haplotype network of the genetic structure amongst 51 Polish ERBV sequences showed a strong correlation between ERBV sequence and individual horses, as well as the stud of origin (Table 4, Figure 5).

## 4. Discussion

The aim of the current study was to determine the frequency of ERAV and ERBV infection among horses at Polish national studs. Some of the horses at five of the studs experienced respiratory disease of varying severity at the time of sampling, while horses at the remaining eleven studs were clinically healthy. The detection of respiratory pathogens in nasal swabs of clinically affected horses helps with the establishment of the potential cause of disease. The awareness of the frequency of virus shedding from healthy horses in a given population is important for preventing the unintentional spread of these viruses within the facility. In addition, it forms a baseline for evaluating the role of such infections during subsequent outbreaks of respiratory disease.

Only one out of 68 nasal swabs from horses with respiratory disease tested positive for ERBV and none were positive for ERAV. This suggests that equine rhinitis viruses were unlikely to be aetiologically involved in the respiratory disease affecting horses at the studs sampled. This is consistent with the low frequency of the molecular detection of equine rhinitis viruses in nasal swabs of horses with sudden onset of respiratory disease in a large USA-based surveillance study. Only 2.3% of 10,296 horses with recent onset of respiratory disease tested between 2008 and 2021 were positive for ERAV or ERBV RNA [17]. In support of this view, experimental infection with either ERAV or ERBV produced only mild or inapparent clinical disease [8,9,18]. However, the detection of either virus from clinically affected horses has also been reported [5,19] and experimental infection with ERAV resulted in the development of overt respiratory disease when the virus was administered in combination with corticosteroids to simulate stress [20]. Subsequent reports from the USA described an apparent increase in the frequency of detection of ERBV from clinically affected horses to approximately 5% [21,22], suggesting that the role of ERBV in equine respiratory disease may need to be re-evaluated.

The lack of the detection of equine rhinitis viruses from diseased horses could have also reflected the timing of sample collection. It is generally considered that the collection of samples shortly after infection provides the best chance of detecting respiratory viruses. The samples from the diseased horses in the current study were collected by attending veterinarians, and hence the horses were sampled at various times following the onset of respiratory disease. However, horses experimentally infected with ERAV shed the virus in their nasal secretions for up to 3 weeks or possibly longer, but the shedding was most consistent within the first week following infection [8,20]. Similarly, ERBV was recovered in one study for up to 11 days post-infection, although the shedding was intermittent in one of the two experimentally infected horses [23]. The virus subsequently spread from experimentally infected horses to naïve horses following their re-introduction to the herd weeks after experimental infection, indicating the development of a carrier state in at least one of the horses [23]. Hence, the varied timing of sample collection in the current study is unlikely to be the sole reason behind the lack of the detection of equine rhinitis viruses from the majority of diseased horses. The processing of samples is unlikely to have negatively affected the results either, as gamma herpesviruses were identified from a large proportion of those samples that had been tested previously for the presence of herpesviral DNA [13].

Equine rhinitis B virus was detected in nasal swabs from 10/553 (1.8%) healthy horses at five different studs, with one to three positive horses identified at each stud. This suggests that ERBV was circulating at those studs without causing overt clinical disease, and is consistent with the high (70.5%) seroprevalence of antibodies to ERBV among 650 horses from the southeastern region of Poland reported previously [12]. Others reported similar results, with detection rates of ERBV in nasal secretions among healthy horses ranging from 0% to 3% [24,25,26,27]. The populations tested included horses at shows [25,26], horses recently imported to the USA [27] or those presented for routine dental care [24]. In contrast to the above studies, Stout and others [28] reported the detection of ERBV in faecal samples from 38/97 (39%) healthy horses at a multi-day show in the USA, suggesting that gastrointestinal infection with presumably acid-stable variants of ERBV may be more common than respiratory tract infections among some horse populations. However, few authors attempted to detect equine rhinitis viruses in faecal samples. None of the samples tested were positive for ERAV in the same study [28].

The lack of detection of ERAV in the current study, as well as in previously reported studies [24,25,26,27], is somewhat puzzling, as ERAV infection is considered common among various horse populations, with seroprevalence increasing with age to above 60% in many studies [11,29,30]. Infection with ERAV has been reported to be most common at the time when young horses enter training facilities [30]. In one study, 75% of Standardbred racehorses that developed respiratory disease during a 41-day observation period at the Canadian training facility showed seroconversion to ERAV, with the highest rate observed among yearlings [31]. In a similar study at the Thoroughbred training facility, 55% of 21 2-year-olds seroconverted to ERAV, which was associated with clinical respiratory disease and subsequent failure to race [32]. Hence, it may be that ERAV did not circulate at any of the studs at the time of sampling, as all the horses sampled were either unbroken youngsters or breeding mares. This is, however, difficult to reconcile with the 72% prevalence of ERAV antibodies among 353 Polish horses of various ages, breeds and locations reported previously [11]. The sources of samples in that study included horse breeding studs, but the exact number of horses from this source was not provided. The attempts to detect ERAV in nasal swabs from a small subset of horses in the same study was unsuccessful, similar to our results. Unfortunately, blood samples were not collected as part of the current study and, therefore, the ERAV/ERBV antibody status of the sampled horses is unknown.

It is also possible that ERAV circulated among the horses sampled, but active infections were not detected due to the type (nasal swab) of samples collected. High ERAV loads were excreted in urine of experimentally infected horses for at least 37 days post-infection and 50/215 (23.3%) urine samples collected post-race from Thoroughbred horses were positive for ERAV in one study [8]. Others also showed a higher rate of ERAV detection by combined RT-PCR and virus isolation in urine (32%) compared with nasal swabs (11.6%) [7].

The 3DPol and 5′ UTR sequence amplified in the current study has been previously shown to be highly conserved between different erboviruses [15]. Perhaps not surprisingly, Polish ERBV sequences were similar to those circulating in other parts of the world, with no correlation between sequence and country of origin.

To our knowledge, this is the first study where the variability of ERBV quasispecies was investigated. ERBV sequences obtained from the same horse were homogeneous and more similar to each other than to sequences from a different horse. As horses were sampled only once, the stability of ERBV sequence over time remains to be determined. It also remains to be established whether some genotypes are more likely than others to establish persistent infection in the horse. Our approach of sequencing individual clones provides an alternative way to address this question when the cost of whole genome sequencing is prohibitive. The availability of longitudinal samples, ideally from both nasal secretions and urine, would be needed to investigate this further.

A relatively strong correlation between genetic structure and the studs from which samples were collected suggests that there were barriers to ERBV spread between studs. This is consistent with the distance between these studs of several hundred kilometres, with the closest studs (VI and VII from Wielkopolska province) separated by more than 50 km.

Viral sequences from three horses from stud VI clustered together in the phylogenetic tree and were also closely linked in the haplotype network, suggesting that they represent the spread of one variant of the virus among horses at the stud. Similarly, viral sequences from three horses from stud XII were also relatively closely related. In contrast, two ERBV sequences obtained from Polish Koniks at stud XVI clustered separately from each other in the ML tree and were positioned in different parts of the network. This may represent the circulation of ERBVs from different sources at that stud. Alternatively, it may reflect differences in the individual virus–host adaptation of ERBV in a primitive breed (Konik Polski) compared with modern breeds, but more virus-positive samples would be needed to test this hypothesis. Unfortunately, only one to three horses from each stud tested positive for ERBV RNA. The low number of viruses detected from each ERBV-positive stud precludes any solid conclusions on the genetic similarities of viruses from the same stud to be made.

In summary, we have presented epidemiological data regarding the frequency of equine rhinitis virus infections among Polish horses at selected national studs, including the first detection of ERBV in Poland. The molecular analysis of the available ERBV sequences suggests that while the 3Dpol sequence may be useful for tracing the origin of viruses at the local level, it is unlikely to be a reliable epidemiological tool for monitoring the spread of ERBV over larger geographical regions.

## Figures and Tables

**Table 1:** Description of horses (*n* = 621) sampled on one occasion between April 2014 and September 2018 at each of the 16 Polish studs included in the study.

Stud Farm	Breed	Horses Sampled [*n*]	Respiratory Disease [*n*]	Region	Sampling Date
I	Arabian	31	31	Lubelskie	Sep 2018
II	Arabian	19	19	Swietokrzyskie	Apr 2015
III	Arabian	7	7	Lubelskie	Feb 2016
IV	Hucul horse	42	0	Malopolskie	May 2018
V	Malopolska horse	42	0	Opolskie	Apr 2016
VI	Wielkopolska horse	47	0	Wielkopolskie	May 2016
VII	Wielkopolska horse	42	0	Wielkopolskie	May 2016
VIII	Wielkopolska horse	30	0	Warminsko-mazurskie	Apr 2016
IX	Silesian horse	44	0	Dolnoslaskie	Apr 2016
X	Thoroughbred	60	1	Mazowieckie	Apr 2016
XI	Thoroughbred	10	10	Wielkopolskie	Apr 2014
XII	Polish Coldblood horse	49	0	Kujawsko-pomorskie	Apr 2016
XIII	Polish Konik	60	0	Warminsko-mazurskie	Jun 2015
XIV	Polish Konik	40	0	Wielkopolskie	May 2017
XV	Polish Konik	50	0	Wielkopolskie	May 2016
XVI	Polish Konik	48	0	Lubelskie	May 2016
Total		621	68		

**Table 2:** Primers and probes used for detection of equine rhinitis virus A (ERAV) and B (ERBV) among horses in Poland.

Assay	Virus	Region	Primers/Probes (5′ to 3′)	Size (Base Pairs)	Reference
Quantitative RT-PCR	ERAV	5′ untranslated region	Forward: AGCGGCKTGCTGGATTTTC	60	[14]
			Reverse: CATYTGYCAGCTTGGTGACA		
			Probe: FAM-CGGTGCCATTGCT-TAMRA		
	ERBV1		Forward: CCCCTTCCCTGAAGATTGCT	61	
			Reverse: GGCAAACGACCAACACATCA		
			Probe: FAM-TTCTTCCAACTAAACCC-TAMRA		
	ERBV2		Forward: CCCCAACCCTTGAGATTGCT		
Conventional RT-PCR	ERBV	3D polymerase–3′ untranslated region	Forward (outer): TTTTGATGCTTCACATTCTCC	782	[15]
			Reverse (outer): CGCTGTACCCTCGGTCCTACTC		
			Forward (inner): CTTACTAYGAATGTGARGGGGC	623	
			Reverse (inner): GCCTCGGCGAGTGAAGAG		

**Table 3:** Number of clones sequenced from each horse and percentage nucleotide identity of clones analysed in the current study.

Strain ID	Stud Farm	No. of Clones Analysed	% Nucleotide Identity	
			Within Clones	Among Horses
PL_ERBV_II_h1	II	5	99.0–99.8	-
PL_ERBV_VI_h1	VI	5	99.5–100	97.1–100
PL_ERBV_VI_h2		4	99.1–99.6	
PL_ERBV_VI_h3		4	99.0–100	
PL_ERBV_VII_h1	VII	5	98.7–100	-
PL_ERBV_VIII_h1	VIII	5	99.1–99.6	-
PL_ERBV_XII_h1	XII	5	98.7–99.6	96.7–99.8
PL_ERBV_XII_h2		4	99.3–99.8	
PL_ERBV_XII_h3		5	99.3–99.6	
PL_ERBV_XVI_h1	XVI	5	99.0–99.6	95.0–99.6
PL_ERBV_XVI_h2		4	99.3–99.6	

**Table 4:** Analysis of molecular variance (AMOVA) results indicating strength of correlation between population genetic structure within Polish equine rhinitis B viruses and selected traits based on partial sequence of 3D polymerase and 3′ untranslated region.

Test	Variation within Populations	Variation among Populations	Fixation Index (PhiST)	*p*
International network				
Geographical region ^1^	96.5%	3.5%	0.03	0.021
Polish network				
Individual horse	2.3%	97.7%	0.98	<0.001
Stud ^2^	28.3%	71.7%	0.72	<0.001

^1^ Australia; Poland; Switzerland; United Arab Emirates; USA. ^2^ II; VI; VII; VIII; XII; XVI.

## Data Availability

The Polish ERBV sequences obtained in this study have been deposited in GenBank under accession numbers PP839207–PP839257.
